# Association of the Val66Met Polymorphism of the BDNF Gene with the Depression in a Mexican Population with Multiple Sclerosis

**DOI:** 10.3390/life15020213

**Published:** 2025-01-31

**Authors:** Brenda Viridiana Rabago-Barajas, Miguel Ángel Macías-Islas, Ana Miriam Saldaña-Cruz, Jesús Emmanuel Arana-Yepez, Eva Maria Olivas-Flores, Adriana Aguayo-Arelis

**Affiliations:** 1 Department of Applied Psychology, University Center for Health Sciences, University of Guadalajara, Guadalajara 44340, Jalisco, Mexico; brenda.rabago@academicos.udg.mx; 2 Department of Neurosciences, University Center for Health Sciences, University of Guadalajara, Guadalajara 44340, Jalisco, Mexico; miguel.mislas@academicos.udg.mx; 3 Institute of Experimental and Clinical Therapeutics, Department of Physiology, University Center for Health Sciences, University of Guadalajara, Guadalajara 44340, Jalisco, Mexico; ana.saldanac@academicos.udg.mx; 4 Pharmacology and Behavior Laboratory, Neuroscience Institute, University Center for Biological and Agricultural Sciences (CUCBA), University of Guadalajara, Guadalajara 44130, Jalisco, Mexico; jesus.arana1293@alumnos.udg.mx; 5 Department of Anesthesiology, Specialty Hospital, National Medical Center of the West, IMSS, Guadalajara 44340, Jalisco, Mexico; eolivasflores@gmail.co

**Keywords:** psychiatric disorder, depression, multiple sclerosis, brain-derived neurotrophic factor, polymorphism

## Abstract

Multiple sclerosis (MS) is a chronic, autoimmune pathology that affects the nervous system. It is characterized by inflammatory lesions that cause axonal damage with neurodegeneration. The signs and symptoms present in this pathology include among others, psychiatric disorders. In MS, depression is the most frequent psychiatric disorder, with prevalence levels of 40 to 60%; to date, the cause is unknown. The brain-derived neurotrophic factor (BDNF) is a neurotrophin related to neuroplasticity. The single-nucleotide polymorphism Val66Met, encoded by the *BDNF* gene, has been associated with various effects, including the presence of neuropsychiatric disorders. The purpose of our study was to evaluate the association between the *BDNF* Val66Met polymorphism and depression in MS patients. Methods: Study design, cases, and controls: Mexican mestizo MS patients. Cases: Patients diagnosed with depression. Controls: Patients without depression diagnosis. Measurements: For depression, the Beck Depression Inventory; for polymorphism, real-time PCR. Results: No statistically significant differences were found in sociodemographic and disease variables between the case and control groups. qPCR analysis showed that 68% of the participants were Val/Val wild-type homozygotes, 29% were Val/Met polymorphism heterozygotes, and 3% were Met/Met polymorphism homozygotes. The presence of the BDNF gene rs6265 polymorphism was associated with a 5.6-fold increase in the probability of depression in the cases compared to the controls. Conclusions: The BDNF Val66Met Polymorphism is associated with depression in Mexican mestizo patients diagnosed with MS.

## 1. Introduction

Multiple sclerosis (MS) is a chronic autoimmune disease characterized by the demyelination of the central nervous system (CNS), specifically affecting the brain and spinal cord. This condition leads to the formation of inflammatory lesions known as myelin plaques, which, over time, initiate a degenerative process [1,2]. The white matter of the CNS is the primary site of damage in MS, resulting in a wide range of neurological symptoms. The most common manifestations of the disease are motor and sensory impairments, such as weakness, spasticity, numbness, and sensory loss. However, because the demyelinating lesions often occur in different areas of the brain, cognitive deficits and psychiatric symptoms are also frequently observed. It is estimated that between 40 and 60% of MS patients experience one or more neuropsychiatric symptoms during the course of the disease, including depression, anxiety, and other affective disturbances [3,4].

Psychiatric manifestations in MS are diverse, with mood disorders being among the most prevalent. Patients frequently present with depression, anxiety, and, less commonly, bipolar disorder, emotional lability, and pseudobulbar affect [5,6]. Depression, in particular, is recognized as the most frequent psychiatric complication, affecting approximately 40–60% of MS patients [7]. Despite its high prevalence, the underlying cause of depression in MS remains unclear. Various theories have been proposed, including the neuroinflammatory hypothesis, which suggests that chronic inflammation in the CNS contributes to the pathophysiology of depression in MS [8,9]. Another significant theory, the neurotrophic hypothesis, implicates brain-derived neurotrophic factor (BDNF) in the development of depression. According to this hypothesis, reduced levels of BDNF, a protein crucial for the survival and differentiation of neurons, may contribute to depressive symptoms [10].

BDNF plays a key role in neuroplasticity, synaptic function, and the overall health of neurons. It is encoded by the BDNF gene, which is part of the neurotrophin family. In recent years, genetic studies have identified several polymorphisms within the BDNF gene, one of the most studied being the Val66Met polymorphism. This polymorphism involves a single-nucleotide substitution at codon 66, where a guanine (G) is replaced by adenine (A), leading to the replacement of valine (Val) with methionine (Met) in the BDNF protein structure [11]. Single-nucleotide polymorphisms (SNPs) like Val66Met are common genetic variations that occur in more than 1% of the population. Although the presence of a polymorphism does not necessarily lead to clinical symptoms, certain SNPs can predispose individuals to various conditions.

The BDNF Val66Met polymorphism significantly interacts with childhood adversity and stressful life events, contributing to the development of depression. Several critical BDNF signaling pathways are closely linked to depression, which can be categorized based on key proteins, such as the tyrosine kinase receptor (TrkB), the p75 neurotrophin receptor (p75NTR), and nuclear factor kappa-B (NF-κB). Notably, the TrkB receptor, a high-affinity receptor for BDNF, plays a central role in BDNF signaling and is involved in neuronal differentiation, cell survival, synaptic plasticity, neurotransmission, long-term potentiation, and neurogenesis. These functions are mediated through the activation of pathways such as PLCγ/PKC, PI3K/Akt, and Ras/Erk. Dysfunction in the BDNF/TrkB signaling pathway reduces tyrosine phosphorylation of TrkB receptors in the brain, diminishing BDNF activity and resulting in depression-like symptoms [12,13,14]. Other pathways, such as the mammalian target of rapamycin (mTORC1) signaling pathway and nuclear factor kappa-B (NF-κB), also play roles in BDNF-associated depression [15]. Recent studies highlight these signaling pathways as critical therapeutic targets for depression treatment [16]. These mechanisms may be disrupted in MS patients, potentially increasing their susceptibility to developing depression [17,18].

BDNF is associated with serotonin imbalance and stress-related conditions, such as hypothalamic–pituitary–adrenal (HPA) axis dysfunction and oxidative stress. This suggests that therapies aimed at increasing BDNF levels could be beneficial for treating depression [19]. Both preclinical and clinical studies have implicated impaired BDNF signaling through its TrkB receptor in the pathophysiology of mood disorders, including depression. Consequently, it appears essential for antidepressants to exert their therapeutic effects by targeting BDNF-TrkB signaling. Additionally, activating this pathway can promote neurogenesis and synaptic plasticity, which support the recovery of neuronal function in individuals with depression [20].

Numerous studies have investigated the relationship between BDNF levels and depressive symptoms in the general population. It has been suggested that individuals with lower serum levels of BDNF are more prone to developing depression [21,22]. Additionally, research has shown that the Val66Met polymorphism may be linked to a heightened vulnerability to a range of psychiatric disorders, including depression, schizophrenia, and bipolar disorder [23]. Given the importance of BDNF in neuronal health, it is hypothesized that the Val66Met variant could influence the development of neuropsychiatric symptoms in MS patients, particularly depression. Despite this, studies specifically focusing on the role of the Val66Met polymorphism in MS-related depression remain scarce.

Currently, no studies have been published analyzing the relationship between the Val66Met polymorphism of the BDNF gene and MS in Latin American populations, let alone its association with depression. Most research to date has focused on European and Asian populations [24,25].

This lack of data in mestizo populations underscores the critical need for research that explores these genetic associations in diverse ethnic groups, particularly in regions like Latin America, where genetic composition and environmental factors may differ substantially from those previously studied. Addressing this gap is essential to provide a more comprehensive understanding of these conditions and to develop targeted interventions that consider the unique genetic and environmental characteristics of underrepresented populations.

In Mexico, research on the Val66Met polymorphism and its potential association with depression in MS patients is particularly limited. To date, no studies have been published analyzing this genetic variation in Mexican populations in the context of MS [26]. Considering the high prevalence of both MS and depression, investigating this relationship in different ethnic groups is of significant clinical interest. The present study aims to address this gap in the literature by conducting a case–control study on Mexican mestizo subjects. Our objective is to evaluate the potential association between the Val66Met polymorphism and depression in MS patients within this population. We hypothesize that the presence of the Val66Met polymorphism may be correlated with an increased risk of depression in MS patients, which could have important implications for understanding the genetic factors contributing to psychiatric symptoms in this disease.

In summary, the investigation into the relationship between genetic factors, such as the BDNF Val66Met polymorphism, and psychiatric manifestations in MS could provide new insights into the underlying mechanisms of depression in this complex disease. By focusing on a Mexican cohort, our research aims to contribute valuable data to the global understanding of MS-related depression and potentially inform future therapeutic strategies tailored to genetic profiles.

## 2. Materials and Methods

### 2.1. Study Design, Population, and Ethics

A case-control study was conducted. Sixty-three Mexican mestizo (according to the criteria of the National Institute of Anthropology, Mexican mestizos are considered as those individuals born in Mexico, with a family that has lived in the country for at least three generations and whose surnames are of Spanish origin) [27] patients diagnosed with MS according to the 2010 McDonald criteria. This study was conducted in the University Center for Health Sciences, and it was approved by the Ethics Committee of the Institute of Experimental and Clinical Therapeutics of the University Center for Type of the Paper Health Sciences CEI/486/2019. Informed consent was signed by all participants.

The inclusion criteria for this study were as follows: being Mexican mestizo, aged 18 years or older, having a diagnosis of MS according to the 2010 McDonald criteria, having been diagnosed with MS for more than one year, having a diagnosis of depression, and having signed the informed consent form. On another hand, for the control group, the same criteria were applied, except for the diagnosis of depression according to the DSM-V. Meanwhile, the exclusion criteria included having a severe visual or auditory deficit, experiencing a relapse in the last 30 days, receiving corticosteroid treatment in the last 30 days, or presenting a psychiatric comorbidity. Finally, the elimination criteria included withdrawal of informed consent or degraded samples that prevented the analysis of polymorphism.

A total of 72 subjects were initially evaluated for this study. Of these, 5 were excluded due to a relapse date of less than 30 days from the study period, and 2 were excluded because their MS diagnosis date was less than one year from the study period. Therefore, the final analysis included 65 participants diagnosed with MS.

### 2.2. Procedures and Instruments

Individuals with a diagnosis of multiple sclerosis were identified, and they attended an evaluation with the clinical team, which included a neurologist, psychologist, and pharmaceutical chemical biologist. The subjects who met the criteria visited the facilities of the University Center for Health Sciences. They were evaluated by the neurologist to confirm the diagnosis of MS. Subsequently, a data collection format and mood scale were applied, and sample extraction was performed. The samples were processed and stored prior to genetic analysis. The data collection format was designed to obtain the main sociodemographic and disease variables such as age, sex, education, type of multiple sclerosis, and years of disease evolution. The Beck Depression Inventory was used and a psychological evaluation was performed by an expert psychologist, and the main consideration was the presence of symptoms over a period of at least 2 weeks prior to the evaluation date. At the time of the assessment, efforts were made to verify that the participants had not experienced a relapse or had corticosteroids treatments over a period equal to or less than 30 days, thus preventing interference with their symptoms. The instruments used are described below.

### 2.3. Physical and Neurological Examination

A neurological examination was performed by an expert neurologist to confirm the diagnosis of MS and determine the score on the Kurtzke Expanded Disability Status Scale (EDSS) [17].

### 2.4. Neurological Examination

Neurological examination is the main clinical tool used in medicine to verify the presence of signs and symptoms by affectation of the nervous system. This examination includes a detailed neurological medical history, an evaluation of mental capacity and emotional state, and an assessment of the function of the twelve cranial nerves. Additionally, it covers coordination, balance, gait, reflexes, sensory function, and the autonomic nervous system.

### 2.5. Kurtzke Expanded Disability Status Scale

This is a scale used globally that is based on neurological examination to quantify the symptoms and measure the progression of MS over time. It verifies eight functional systems such as pyramidal, sensory, vesical, intestinal, motor, visual, cerebellar, brainstem, and cognition functions. The EDSS has proven to be an adequate tool capable of describing and quantifying a person’s health status based on their signs and symptoms and the possible progression of the disease by analyzing the possibility of disability [28].

### 2.6. Psychological Examination

A specialized psychologist conducted the psychological evaluation to determine the presence or absence of depression using the criteria established in the fifth edition of the Diagnostic and Statistical Manual of Mental Disorders, DSM-5 [29], following an initial interview, during which data on the patient’s mood were collected through the Beck Depression Inventory. This self-administered scale measures the presence and severity of depression through 63 questions addressing major depressive symptoms. Each question provides four possible responses, reflecting varying degrees of symptom severity. The questions are organized into 21 categories that represent behavioral and somatic symptoms of depression, based on a specific timeframe. A score higher than 13 points [30] combined with the clinical evaluation results from the psychologist was required for a diagnosis of depression.

In contrast, a patient with multiple sclerosis without depression would score 12 points or less on the Beck Depression Inventory and would not meet the DSM-5 criteria for depression during the psychological evaluation. These patients are expected to display a stable mood, an absence of persistent feelings of sadness or hopelessness, and no significant behavioral or somatic symptoms associated with depression, such as sleep disturbances, fatigue beyond what is typically experienced due to multiple sclerosis, or a loss of interest in activities. The clinical evaluation would corroborate these findings, indicating no significant psychological distress or impairment attributable to depressive symptoms.

### 2.7. Genetic Analysis

Peripheral venous blood samples were collected by venipuncture for genetic analysis. The procedures were followed for the treatment prior to storage of the samples, which were refrigerated at a temperature between a minimum of 2 to a maximum of 8 °C.

- DNA extraction.

The DNA extraction was conducted through cell disruption, protein elimination by precipitation with salts at different concentrations, DNA concentration by precipitation with alcohols, washing to eliminate the remains, and resuspension. All of this was performed after a process of standardization of the technique. In addition, a molecular analysis after DNA extraction was performed, and the integrity was verified. We utilized electrophoresis in a 6% polyacrylamide gel stained with silver nitrate and the determination of concentration and purity was performed via spectrophotometry.

- Real-time PCR.

The DNA in optimal conditions was stored at −20 °C until processing. In the genotyping process, the Val66Met polymorphism of the BDNF gene was determined through real-time PCR for allelic discrimination using TaqMan^®^ probes, following the protocols of Taqman ID: C__11592758_10, on Applied Biosystems Step0ne Real Time PCR equipment (Applied Byosystems, Foster City, CA, USA). The PCR cycling conditions were as follows: initial denaturation at 95 °C for 10 min, followed by 40 cycles of denaturation at 95 °C for 15 s and extension at 60 °C for 60 s. Analyses of the genotypes of the DNA samples were performed in duplicate. The presence of wild-type genotypes and polymorphisms of both genes were defined by comparing the relative fluorescence endpoint created by the degradation of each fluorescently labeled probe with TaqMan^®^.

### 2.8. Statistical Analysis

Descriptive statistics, including frequency and percentage, were used to characterize the sample. In addition, the Kolmogorov–Smirnov test was employed to assess the normality of the data. Subsequently, Student’s *t*-test or the Mann–Whitney U Test (according to the distribution of the variables) was used to compare quantitative variables between the cases and controls. Additionally, chi-square was applied to compare the proportions between the study groups. Finally, the allele frequencies and percentages for the total sample were obtained, and the alleles were categorized into val (wild) and met (polymorphic). The association risk was obtained using odds ratio (OR) with a 95% confidence interval.

Analysis was conducted using three models: wild homozygous versus polymorphic heterozygous model (val/val vs. val/met), wild homozygous versus polymorphic homozygous model (val/val vs. met/met), and finally the wild homozygous model versus the sum of the heterozygous and the polymorphic homozygous (val/val vs. val/met + met/met) models. These analyses were performed separately for each group, and the level of significance was set at a *p* value < 0.05.

## 3. Results

**Figure 1:**
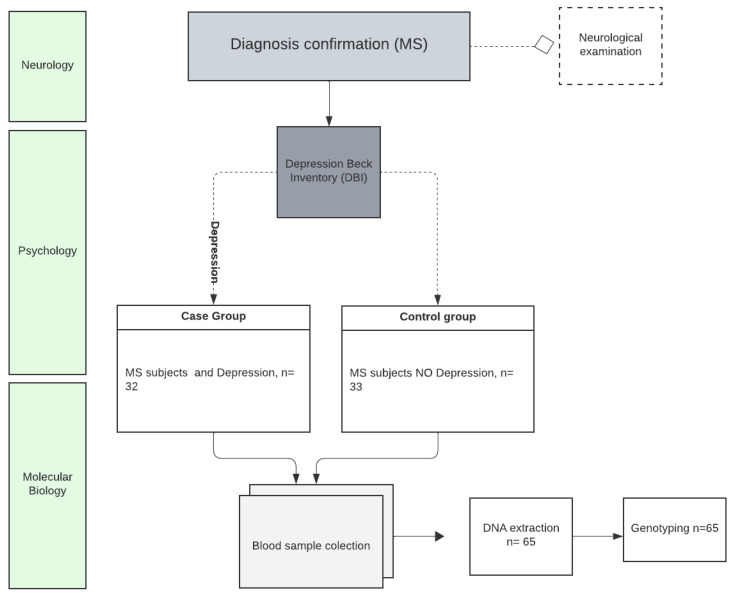
Methodological design.

A total of 72 subjects were initially evaluated for this study. Of these, five were excluded due to a relapse date of less than 30 days, and two were excluded because their multiple sclerosis (MS) diagnosis date was less than one year from the study period. Therefore, the final analysis included 65 participants diagnosed with MS (Figure 1).

These 65 participants consisted of 32 cases (78% women, 22% men) and 33 controls (64% women, 36% men). DNA samples were successfully obtained from all the participants. There were no significant differences between cases and controls in terms of age or education level. Education was measured as the total number of years completed, ranging from primary education to the highest grade achieved or the current grade level.

Moreover, there were no differences between the cases and controls in terms of disease duration or Expanded Disability Status Scale (EDSS) scores. Both groups exhibited comparable characteristics regarding the progression of the disease and the degree of disability (Table 1).

In addition, of the total sample (*n* = 65), 68% had the wild val/val genotype, 29% had the heterozygous genotype, and 3% had the polymorphic homozygous genotype. On the other hand, 82% had the wild allele (val) and 18% had the met allele (Table 2) genotype.

To analyze the relationship between the val66met polymorphism of the BDNF gene and depression in Mexican mestizo patients, we separated those subjects with MS who had depression who were considered the cases and the control group from those subjects with MS without depression. For each group, the presence of polymorphism was assessed, along with whether individuals were in a heterozygous or homozygous state. In the control group (*n* = 32), the predominant genotype was the val/val genotype in 50% of the sample (*n* = 16), while the polymorphic val/met variants were present in 44% (*n* = 14) and 2% (*n* = 6) of the sample, respectively. In the control group, the non-polymorphic genotype was observed in 85% (*n* = 28) of the sample, and the only polymorphic val/met variant was found in 15% of the sample. In terms of the allele frequency, we found that the val allele was present in 72% of the cases (2*n* = 64) and in 92% of the controls (2*n* = 66), respectively, while the met allele was present in 28% of the cases and only in 8% of the controls.

The polymorphic allele was more frequent in the case group (Table 3). Odds ratio (OR) was used to determine the risk. The val/val (non-polymorphic) model versus the val/met polymorphism had an OR of 0.20 (*p* < 0.05). The val/mal model versus the met/met polymorphism had an OR of 4.9 (*p* < 0.05).

Finally, we used an analysis of the val/val model (non-polymorphic variant) versus the sum of the polymorphic variants (val/met + met/met); an OR of 5.6 (*p* < 0.05) was obtained (Table 3). The presence of the Val66Met polymorphism of the BDNF gene in this population of subjects with MS increases the probability of presenting depression by 5.6 times (Table 3).

## 4. Discussion

Multiple sclerosis is an irreversible neurological disorder characterized by axonal damage from autoimmune responses, leading to a range of clinical symptoms. Depression is a common neuropsychiatric symptom in MS, potentially triggered by diagnosis-related stress, referred to as reactive depression [31]. However, there is also consideration of a biological predisposition to depression in MS patients [32]. The neurotrophic hypothesis emphasizes the role of brain-derived neurotrophic factor, a protein essential for neural development, maintenance, and survival in the development of depression [33]. BDNF affects emotional regulation through its effects on the prefrontal cortex and limbic system [34,35] and impacts neurotransmission systems such as the serotoninergic pathway [36,37]. Although the Val66Met polymorphism in the BDNF gene does not inhibit BDNF production, its functional implications, particularly in depression, remain under investigation [38,39].

The aim of this study was to explore the association between the Val66Met polymorphism and depression in patients with MS. Additionally, we analyzed the genotypic and allelic frequencies of this polymorphism in a population of Mexican mestizo patients with MS. Our findings revealed that the wild-type Val/Val genotype was present in 68% of the sample, while 32% exhibited polymorphic variants, consistent with previous studies conducted in MS populations. For example, Nociti et al. (2019) [40] reported similar genotypic distributions in MS patients and highlighted the role of the Val66Met polymorphism as a potential marker of disease progression. Likewise, Santoro et al. (2016) [26] demonstrated the relevance of this polymorphism in MS, particularly in its association with depression and patients’ perceptions of disease severity [15,40].

Interestingly, the distribution of the Val66Met polymorphism in our study aligns with the genotypic and allelic frequencies reported in European MS populations, suggesting that, despite genetic diversity between populations, this polymorphism may have similar prevalence particularly among Spaniards, demonstrating the profound influence of European ancestry in our group [41]. However, certain population-specific variations cannot be ruled out, especially given the mestizo population’s unique genetic background, which is characterized by the admixture of European, Indigenous, and African ancestry.

Our findings also align with Nociti et al. (2019) [40], who proposed that epigenetic modifications in the BDNF gene, including methylation patterns associated with the Val66Met polymorphism, could be indicative of disease progression and symptom severity in MS.

Our study contributes to understanding the genetic predisposition to psychiatric symptoms in MS. Specifically, our findings add to the body of evidence suggesting that the Val66Met polymorphism may influence neuropsychiatric outcomes, such as depression, by modulating BDNF availability and function. This is particularly relevant given the role of BDNF in neuronal plasticity, emotional regulation, and stress response [42,43].

Our results showed that the frequency of the Met allele was 18%, consistent with data reported in a Spanish MS patient cohort, which found a frequency of 20% for this allele Blanco et al. (2006) [44]. This similarity suggests that the prevalence of the Met allele in MS patients may be relatively consistent across different populations. In comparison with other neurodegenerative diseases, higher Met allele frequencies have been observed. For instance, Cagni et al. (2017) [45] reported a frequency exceeding 25% in a Brazilian population with Parkinson’s disease, while meta-analyses, such as those by Wang et al. (2019) [46] and Mariani et al. (2015) [47], highlight the relevance of this polymorphism in Parkinson’s disease, particularly in its association with cognitive impairment and mood disorders.

The Met allele has been proposed to influence susceptibility to neurodegenerative diseases by affecting BDNF function. BDNF plays a critical role in neuronal plasticity, survival, and synaptic modulation, all of which are essential in maintaining neural integrity. The presence of the Met allele has been linked to altered intracellular trafficking and reduced activity-dependent release of BDNF, which may exacerbate neuronal vulnerability in conditions such as MS and Parkinson’s.

These findings underscore the potential importance of the Met allele not only in MS but also in the broader context of neurodegenerative diseases. The consistency between our results and those from studies in other populations and diseases reinforces the idea that the Met allele may be a critical genetic factor influencing disease susceptibility and progression. Further research could investigate the functional implications of this allele in MS, particularly its relationship with clinical outcomes such as cognitive impairment and psychiatric symptoms. Such studies could also explore whether the interplay between the Met allele and environmental or epigenetic factors contributes to disease variability in different populations [45,46,47,48].

When analyzing the relationship between the Val66Met polymorphism and the presence of depression, several studies have highlighted its strong association with the severity of clinical symptoms in individuals with psychiatric disorders such as bipolar disorder and schizophrenia. For example, Numata et al. (2006) [49] found that this polymorphism is significantly linked to the age of onset and the manifestation of symptoms in patients with schizophrenia. Similarly, Sun et al. (2013) [50]. reported an association between the Val66Met polymorphism and the presence of anxiety and depression symptoms in individuals with schizophrenia, particularly within the Chinese Han population. These findings suggest that the Val66Met polymorphism contributes to the development of psychiatric symptoms, including anxiety and depression, across various populations and conditions [49,50].

In the present study, we observed that the Val66Met polymorphism increases the risk of developing depression in patients with multiple sclerosis (MS) by 5.6-fold. This finding aligns with the broader body of evidence suggesting that brain-derived neurotrophic factor (BDNF) plays a critical role in the pathophysiology of depression. The presence of this polymorphism appears to amplify the susceptibility to psychiatric symptoms not only in the general population but also in individuals with MS, further underscoring its clinical relevance. These results contribute to the growing understanding of the molecular mechanisms underlying depression and suggest that targeting BDNF-related pathways could hold promise for therapeutic interventions.

Identifying depression in MS patients based solely on the Val66Met polymorphism in the BDNF gene presents considerable challenges. Depression is a multifactorial condition arising from a complex interplay of genetic, environmental, and psychological factors. Extensive research has explored genetic polymorphisms associated with depression to identify DNA variations that may increase susceptibility to this disorder. Among the most commonly studied are polymorphisms in genes involved in the regulation of neurotransmitters such as serotonin, dopamine, norepinephrine, and cortisol, all of which play critical roles in mood regulation and stress response. For instance, studies have shown that the 5-HTTLPR polymorphism in the serotonin transporter gene is associated with an increased risk of depression, as evidenced in patients with Parkinson’s disease Similarly, variations in the dopaminergic system, such as the COMT Val108/158Met and DRD4 polymorphisms, have been linked to mood disorders, anxiety symptoms, and stress responses [51,52,53].

It is important to emphasize that while certain polymorphisms, including Val66Met, may increase the risk of developing depression, their effects are generally moderate and strongly influenced by interactions with environmental and social factors. For example, Alexopoulos et al. (2021) [51] demonstrated that the effects of genetic polymorphisms, such as BDNF Val66Met and COMT Val108/158Met, on depressive and anxiety symptoms are modulated by situational stressors, such as those experienced during military training. Moreover, Konishi et al. (2014) [53] highlighted that gene–gene and gene–gender interactions play a pivotal role in modulating the risk of psychiatric conditions, further illustrating the complexity of genetic contributions to depression.

These findings underscore the need for a holistic approach to understanding depression in MS patients, considering not only genetic predisposition but also the intricate interplay of environmental and psychosocial factors. While the Val66Met polymorphism may contribute to increased susceptibility, a comprehensive assessment of additional genetic, environmental, and individual factors is essential to fully understand and address the mechanisms underlying depression in this population.

In summary, our study suggests a higher risk of depression associated with the polymorphism compared to previously reported studies. One notable aspect, when compared with other studies, was the number of patients included. However, we considered that the sample has been sufficient to find important results, mainly due to the association of the polymorphism with the presence of depression. In the same way, this is the first study carried out in patients with MS that has found an association between the Val66Met polymorphism of the BDNF gene and depression. Although our methodological design does not prescribe a therapeutic intervention for depression in MS, our study provides a precedent for understanding the prevalence of this polymorphism within our mixed-race Mexican population and its implications for depression in MS patients.

Furthermore, our findings contribute to the knowledge of depression in multiple sclerosis. We recommend further longitudinal studies involving patients with the polymorphism to elucidate whether its presence in the Val66Met polymorphism can predict depression and potentially its severity throughout the course of MS progression. It is possible that the behavior of this polymorphism in relation to the functioning of the central nervous system is different for Mexican mestizos compared to other races, particularly in individuals diagnosed with MS.

However, this is the first study carried out in Mexican mestizo patients with multiple sclerosis. Given the scarcity of findings in mestizo populations due to the fact that most published studies focus on Caucasian or Asian populations, this work highlights the critical need to address this gap. While this study does not aim to isolate the interplay of factors in depression, it underscores the importance of considering contextual elements such as social climate and access to healthcare. These factors represent distinct challenges in Latin American countries, differing significantly from those explored in studies conducted in other regions.

Studies with small sample sizes, such as this one, are invaluable for exploring genetic predispositions or disadvantages in the face of challenging environments or circumstances. Moreover, initiating analyses of other forms of inequality is essential to understanding why some individuals fail to respond to certain treatments. Such investigations pave the way for identifying the underlying factors that may contribute to these disparities, offering a foundation for developing more effective, tailored therapeutic interventions in diverse populations.

## 5. Conclusions

In our study, we found a 5.6% association between the Val66Met genetic polymorphism of the BDNF gene and the risk of developing depression in Mexican mestizo patients with multiple sclerosis. This represents a high probability that, in the presence of the Val66Met polymorphism, a mood disorder such as depression will develop. This study provides valuable information on the role of genetic variability in neurodegenerative diseases and their clinical manifestations. In addition, it provides relevant information for understanding depression in MS patients.

## 6. Limitations

The sample size of 65 participants represents a notable limitation in our study, primarily due to the low prevalence of the disease and the specific polymorphism examined. To address this challenge, we utilized robust statistical analyses and carefully selected inclusion criteria to maximize representativeness within the limitations of our sample. Moreover, the case–control design of this study does not allow for the evaluation of depression progression in relation to the presence or absence of the polymorphism. Furthermore, our study was confined to a regional sample of the Mexican population with multiple sclerosis (MS) from the western part of the country, and we did not include polymorphism data from a healthy control group. We believe that a larger and more diverse sample, including participants from different ethnic and geographical backgrounds, could improve the robustness and generalizability of our findings. In future studies, we aim to address these limitations to achieve more comprehensive and accurate results.

## Figures and Tables

**Table 1:** Baseline sociodemographic and disease characteristics in MS patients.

Variable	Cases (*n* = 32)	Controls (*n* = 33)	*p* Value
**Sociodemographic features**			
Age *	40.22 ± 11.57	39.09 ± 10.76	0.68
Level of education *	12.75 ± 4.44	14.15 ± 3.31	0.24
Female sex (percentage) **	25 (78%)	21 (64%)	0.51
Male sex (percentage) **	7 (22%)	12 (36%)	
**Features of the disease**			
Years of evolution *	9.13 ± 6.63	10.67 ± 6.48	0.34
EDSS *	3.5 ± 1.61	3.2 ± 1.89	0.24
Annualized relapse rate *	0.49 ± 0.70	0.43 ± 0.63	0.21

Cases: Patients with a diagnosis of MS plus depression. Controls: Patients with a diagnosis of MS without depression. EDSS: Expanded Disability Status Scale. Statistical tests: Data are shown as mean and standard deviation. Qualitative variables are expressed as frequencies. * T for Student’s *t*-test. ** Chi-square; *p* ≤ 0.05.

**Table 2:** Gene polymorphism identified in MS patients.

Multiple Sclerosis *n* = 65		
Genotype	Frequency	Percentage
Val/Val	44	68%
Val/Met	19	29%
Met/Met	2	3%
**Alleles 2*n* = 130**		
Val	Wild-Type	107 (82%)
Met	Polymorphic	23 (18%)

Val/Val (homozygous wild genotype), Val/Met (heterozygous genotype), Met/Met (polymorphic homozygous genotype). Data are shown as frequencies and percentages. Frequencies and percentages in alleles. Val (valine), Met (methionine).

**Table 3:** Evaluation of the rs6265 polymorphism as a depression predictor for MS patients.

Multiple Sclerosis (*n* = 65)	Depression (*n* = 32)	Without Depression (*n* = 33)	OR	95% CI	*p* Value
**Genotype**					
VAL/VAL, *n* = 44 (68%)	16 (50%)	28 (85%)	-------	--------	
VAL/MET, *n* = 19 (29%)	14 (44%)	5 (15%)	-------	--------	
MET/MET *n* = 2 (3%)	2 (6%)	0 (0%)	-------	--------	
VAL/VAL versus VAL/MET	--------	--------	**0.20**	**0.06**–**0.67**	**0.006** *
VAL/MET versus VAL/VAL	--------	--------	**4.9**	**1.48**–**16.13**	**0.007** *
**Genetics Models**					
Dominant model (VAL/VAL vs. VAL/MET + MET/MET)	--------	--------	**5.6**	**1.72**–**18.17**	**0.002** *
**Alleles**, **2*n*** = **130**	2*n* = 64	2*n* = 66			
Allele VAL, 2*n* = 104 (%)	46 (72)	61 (92)	**0.20**	**0.07**–**0.60**	**0.002** *
Allele MET, 2*n* = 22 (%)	18 (28)	5 (8)	**1.65**	**1.65**–**13.81**	**0.002** *

OR: odds ratio; CI: confidence interval; Val/Val: wild homozygous genotype; Val/Met: heterozygous genotype; MET/MET: polymorphic homozygous genotype. Statistical significance (*p* < 0.05) is indicated by an asterisk (*) in the table.

## Data Availability

For more information regarding the data availability, please contact the corresponding author via email.
